# Correlations between serum concentration of three bone-derived factors and obesity and visceral fat accumulation in a cohort of middle aged men and women

**DOI:** 10.1186/s12933-018-0786-9

**Published:** 2018-11-13

**Authors:** Yiting Xu, Xiaojing Ma, Xiaoping Pan, Xingxing He, Yunfeng Xiao, Yuqian Bao

**Affiliations:** 1 Department of Endocrinology and Metabolism, Shanghai Jiao Tong University Affiliated Sixth People’s Hospital; Shanghai Clinical Center for Diabetes; Shanghai Key Clinical Center for Metabolic Disease; Shanghai Diabetes Institute, Shanghai Key Laboratory of Diabetes Mellitus, 600 Yishan Road, Shanghai, 200233 China; 2 Department of Radiology, Shanghai Jiao Tong University Affiliated Sixth People’s Hospital, Shanghai, 200233 China

**Keywords:** Osteocalcin, Fibroblast growth factor 23, Neutrophil gelatinase-associated lipocalin, Lipocalin-2, Fat distribution, Visceral fat area

## Abstract

**Background:**

The aim of this study was to investigate the interrelationships between three bone-derived factors [serum osteocalcin (OCN), fibroblast growth factor (FGF) 23, and neutrophil gelatinase-associated lipocalin (NGAL) levels] and body fat content and distribution, in order to reveal the potential endocrine function of bone in the development of obesity.

**Methods:**

We recruited 1179 people (aged 59.5 ± 6.2 years) from communities in Shanghai. Serum OCN levels were determined using an electrochemiluminescence immunoassay. Serum FGF23 and NGAL levels were determined using a sandwich enzyme-linked immunosorbent assay. The abdominal fat distribution, including visceral fat area (VFA), was assessed by magnetic resonance imaging. Visceral obesity was defined as a VFA ≥ 80 cm^2^.

**Results:**

Serum OCN levels were inversely correlated with body fat parameters, while FGF23 and NGAL were positively correlated (*P* < 0.05). After adjusting for confounders, waist circumference (W) and VFA had a closer relationship with serum OCN, FGF23, and NGAL levels than body mass index (BMI) and body fat percentage (fat%, all *P* < 0.05). The risk of visceral obesity significantly increased with higher FGF23 and/or NGAL levels, as well as with reduced OCN levels (all *P* < 0.05). In addition, serum OCN, FGF23, and NGAL levels were independently associated with visceral obesity (all *P* < 0.01). The relationships persisted among subjects with normal glucose tolerance or subjects with hyperglycaemia (both *P* < 0.05).

**Conclusions:**

Compared to the indicators of overall adiposity such as BMI or fat%, visceral adiposity indicators (W or VFA) were more closely related to serum OCN, FGF23 and NGAL levels. There was no interaction among the relationship of three bone-derived factors with visceral obesity, which revealed the independent relationship of endocrine function of skeleton with body fat.

**Electronic supplementary material:**

The online version of this article (10.1186/s12933-018-0786-9) contains supplementary material, which is available to authorized users.

## Introduction

Osteoblasts, comprising 5% of all bone cells, are responsible for the subsequent replacement with new bone after removal of old or damaged bone. Furthermore, osteoblasts give rise to terminally differentiated osteocytes, the most abundant skeletal cell, which are embedded in the bone matrix [1]. Osteoblasts and osteocytes can synthesize and secrete molecules, including growth factors, cytokines and chemokines to maintain the remodeling and architecture of bone. However, based on evidence that has accumulated in the past decade, biologically active factors which have endocrine function are also secreted by osteoblasts and osteocytes, including osteocalcin (OCN) and fibroblast growth factor (FGF) 23 [2, 3]. Bone has recently been reported to be the predominant organ expressing lipocalin-2 (LCN-2), also referred to neutrophil gelatinase-associated lipocalin (NGAL), secreted by osteoblasts and with at least tenfold higher expression levels in bone than in adipose tissue [4]. Bone regulates several important physiological functions, such as mineral homeostasis, inflammatory response and energy metabolism, by releasing the aforementioned hormones [5, 6].

Fat and bone are linked by several pathways. Bone was ever recognized ultimately to serve the function of providing a skeleton appropriate to the mass of adipose tissue it is carrying. In addition to its structural role, bone has also been recognized as an endocrine organ for the hormonal modulation of energy homeostasis, in which bone-derived factors may play an important role [7]. As shown in animal studies, OCN is associated with obesity by regulating systemic glucose and insulin resistance [2]. FGF23 regulates body fat content and distribution, in addition to its function in regulating mineral metabolism [6, 8]. NGAL participates in glucose tolerance, insulin sensitivity, and insulin secretion to maintain glucose homeostasis [4]. Studies in human subjects have also revealed correlations between the levels of these hormones and obesity. Our previous studies reported significant positive correlations between both serum FGF23 and NGAL levels and obesity [9, 10], whereas serum OCN levels were significantly inversely correlated with obesity [11, 12].

However, researchers have not determined whether the relationships between the three bone-derived factors listed above with body fat content and distribution are independent of each other. As the risk of metabolic disorders increases with age and gradually affects the adipose tissue, this study recruited middle-aged and elderly people, and aimed to explore the associations between bone-derived factors and the body adiposity index using an automatic bioelectrical impedance analyser to measure fat mass and magnetic resonance imaging to accurately quantify abdominal subcutaneous fat area (SFA) and visceral fat area (VFA).

## Materials and methods

### Subjects

The study enrolled middle-aged and elderly subjects from communities in Shanghai between October 2015 and July 2016. Subjects were recruited mainly through promotional posters in local community health center or through the acquaintances of study participants. The collected data were derived from standardized questionnaires including information on current and previous illnesses and medications; physical examinations; biochemical measurements; body composition and abdominal fat distribution measurements. The following inclusion criteria were used: subjects aged ≥ 45 years who voluntarily participated in the study and were able to provide the information required for the study. Subjects with a history of diabetes and/or hypoglycaemic therapy, cardiovascular disease, malignancy, hepatic or thyroid dysfunction, and an estimated glomerular filtration rate (eGFR) < 60 mL/min/1.73 m^2^ were excluded. Additionally, subjects receiving steroid hormones or other treatments that would likely affect bone metabolism, as well as subjects receiving any other medications, such as antihypertensive therapy and lipid lowering therapy, were also excluded. Ultimately, 1179 eligible subjects with complete data were recruited for the study.

### Biochemical measurements

Venous blood samples were collected after a 10-h overnight fast. The following biochemical indices were measured: fasting plasma glucose (FPG), glycated haemoglobin A_1c_ (HbA_1c_), fasting serum insulin, serum total cholesterol, triglyceride (TG), high-density lipoprotein cholesterol (HDL-C), low-density lipoprotein cholesterol (LDL-C), C-reactive protein (CRP), serum creatinine, serum calcium (Ca), OCN, FGF23 and NGAL levels. Subjects also provided a 2-h plasma glucose (2hPG) blood sample following a 75-g oral glucose tolerance test. Standard laboratory measurements were performed using previously described methods [12]. The homeostasis model assessment-insulin resistance (HOMA-IR) index was calculated using the formula HOMA-IR = FPG (mmol/L) × fasting serum insulin (mU/L)/22.5. The eGFR was calculated by the Chronic Kidney Disease Epidemiology Collaboration formula [13].

Serum total OCN levels were determined using an electrochemiluminescence immunoassay (Roche Diagnostics GmbH), for which the intra-assay and inter-assay coefficients of variation were 1.2–4.0% and 1.7–6.5%, respectively [14]. Serum intact FGF23 levels were determined using a sandwich enzyme-linked immunosorbent assay kit (Kainos kit, Kainos Laboratories, Inc., Tokyo, Japan), for which the intra-assay and inter-assay coefficients of variation were 5.6% and 8.2%, respectively [9]. Serum NGAL levels were determined using a standard enzyme-linked immunosorbent assay (Antibody and Immunoassay Services, University of Hong Kong, Hong Kong), for which the intra- and inter-assay coefficients of variation were 1.8% and 6.8%, respectively [10].

### Body composition and visceral fat distribution measurements

Height, weight and waist circumference (W) were measured by previously standardized methods [12]. Body mass index (BMI) = weight (kg)/height^2^ (m^2^). According to the 1998 World Health Organization criteria, overweight/obesity was defined as a BMI ≥ 25.0 kg/m^2^ [15]. Body fat percentage (fat%) and total body fat mass (FM) were measured by a bioelectrical impedance analyser (TBF-418B; Tanita Corp., Tokyo, Japan). The visceral fat distribution, including VFA and SFA, was assessed using magnetic resonance imaging (Archiva 3.0 T; Philips Medical Systems, Amsterdam, The Netherlands). Image quality was controlled by an experienced operator. Average values for the VFA and SFA were calculated using Image analysis software (slice-O-matic, version 4.2; Tomovision Inc., Montreal, Quebec, Canada) according to a previously described protocol [12]. A VFA of 80 cm^2^ was applied as the cut-off point for visceral obesity [16].

### Covariates and subgroup

Blood pressure was measured using a standardized method reported elsewhere [12]. Smoking was defined as the use of at least one cigarette per day for at least 6 months [12]. Menopausal status was defined as at least 12 continuous months of amenorrhea in the absence of other medical conditions. Diabetes was diagnosed when FPG ≥ 7.0 mmol/L and/or 2hPG ≥ 11.1 mmol/L; impaired glucose regulation was diagnosed when 6.1 mmol/L ≤ FPG < 7.0 mmol/L and/or 7.8 mmol/L ≤ 2hPG < 11.1 mmol/L [17]. Subjects with diabetes and individuals with impaired glucose tolerance were classified as the hyperglycaemia group.

### Statistical analyses

All statistical analyses were performed using SPSS for Windows software (ver. 20.0; SPSS Inc., Chicago, IL, USA). The normality of the data distribution was determined by the one-sample Kolmogorov–Smirnov test. After a normality test, data with a normal distribution are presented as the mean ± standard deviation, while data with a skewed distribution are presented as medians with interquartile ranges. Categorical variables are reported as numbers with percentages. For continuous data, inter-group comparisons were performed by an independent sample t-test (normal distribution) or the Mann–Whitney U-test (skewed distribution); the Chi squared test was applied to categorical variables. A partial correlation analysis was conducted to determine the correlations between serum OCN, FGF23 and NGAL levels with body fat parameters. The independent correlations between overall and visceral adiposity with serum OCN, FGF23 and NGAL levels were investigated using multivariate linear regression analyses and are reported as differences in the levels of the three bone-derived factors with 95% confidence intervals. Serum OCN, FGF23, NGAL levels and adiposity parameters were standardized to a mean of zero and standard deviation of 1 (based on the study sample distribution) before performing the analysis. A logistic regression analysis was performed to examine the relationship between visceral obesity and the three standardized bone-derived factors. Models were adjusted for age, gender, menopausal status (in women), smoking status, SBP, DBP, HbA_1c_, HOMA-IR, TG, HDL-C, LDL-C, CRP, Ca, and eGFR. All reported *P* values were two-tailed, and *P* < 0.05 was considered statistically significant.

## Results

### Characteristics of the study participants

The study enrolled 1179 subjects with a mean age of 59.5 ± 6.2 years and included 465 men and 714 women. The clinical characteristics of the subjects are shown in Table 1. Among the total population, serum OCN levels were 19.8 (15.9–24.8) ng/mL, serum FGF23 levels were 34.6 (28.3–41.7) pg/mL, and serum NGAL levels were 45.0 (31.4–61.8) ng/mL. The mean BMI was 23.8 ± 3.0 kg/m^2^. The proportion of subjects with overweight/obesity was 27.7%. Subjects with overweight/obesity had significantly lower serum osteocalcin levels and higher serum FGF23 levels than subjects without these conditions (*P* < 0.001). Serum NGAL levels showed an elevated trend in subjects with overweight/obesity (*P* = 0.085, presented in Additional files 1, 2). The median (interquartile range) VFA was 78.2 (54.8–105.7) cm^2^. The proportion of subjects with visceral obesity was 47.8%. Significantly lower serum OCN levels were observed in subjects with visceral obesity compared with subjects without this phenotype [18.3 (14.9–22.5) ng/mL versus 21.2 (16.8–26.1) ng/mL, *P* < 0.01], while serum FGF23 and NGAL levels were significantly elevated in subjects with visceral obesity [33.3 (26.7–40.0) pg/mL versus 36.3 (29.3–44.2) pg/mL, *P* < 0.01; 41.1 (29.6–55.9) ng/mL versus 50.0 (35.4–66.9) ng/mL, *P* < 0.01, respectively].

**Table 1:** Clinical Characteristics of the study subjects

Variables	Men/Women (n = 465/714)
Age (years)	59.5 ± 6.2
BMI (kg/m^2^)	23.8 ± 3.0
W (cm)	83.0 (77.0–89.0)
FM (kg)	17.4 (13.9–21.5)
Fat%	28.2 (22.7–33.9)
SFA (cm^2^)	168.1 (130.5–218.4)
VFA (cm^2^)	78.2 (54.8–105.7)
FPG (mmol/L)	5.7 (5.4–6.1)
2hPG (mmol/L)	7.1 (5.8–8.6)
OCN (ng/mL)	19.8 (15.9–24.8)
FGF23 (pg/mL)	34.6 (28.3–41.7)
NGAL (ng/mL)	45.0 (31.4–61.8)
Smoking, n (%)	241 (20.4)
Overweight/obesity, n (%)	327 (27.7)
Visceral obesity, n (%)	564 (47.8)
Hyperglycaemia, n (%)	546 (46.3)

BMI, body mass index; W, waist circumference; FM, fat mass; Fat%, fat percentage; SFA, subcutaneous fat area; VFA, visceral fat area; FPG, fasting plasma glucose; 2hPG, 2-h plasma glucose; OCN, osteocalcin; FGF23, fibroblast growth factor 23; NGAL, neutrophil gelatinase-associated lipocalin

### Body fat parameters affecting the levels of bone-derived factors

According to the partial correlation analysis adjusted for age, gender, and menopausal status, serum OCN levels were negatively correlated with BMI, W, FM, fat%, and SFA (*P* < 0.01), and, in particular, were significantly correlated with VFA (r = − 0.185, *P* < 0.01). Serum FGF23 levels were positively correlated with BMI, W, FM, fat%, and SFA (*P* < 0.01), as well as VFA (r = 0.148, *P* < 0.01). Additionally, serum NGAL levels were positively correlated with W, FM, fat% (*P* < 0.05), and particularly VFA (r = 0.182, *P* < 0.01). Serum OCN levels were negatively correlated with serum NGAL levels (r = − 0.077, *P* = 0.009). Neither serum OCN levels nor serum NGAL levels were correlated with serum FGF23 levels (r = − 0.041, *P* = 0.159; r = − 0.041, *P* = 0.163, respectively, see Table 2).

**Table 2:** Partial correlations of serum OCN, FGF23 and serum NGAL levels with body fat parameters

Variables	OCN	FGF23	NGAL
BMI	− 0.176**	0.125**	0.049
W	− 0.187**	0.133**	0.094**
FM	− 0.189**	0.152**	0.075*
Fat%	− 0.184**	0.142**	0.064*
SFA	− 0.112**	0.106**	0.027
VFA	− 0.185**	0.148**	0.182**

OCN, osteocalcin; FGF23, fibroblast growth factor 23; NGAL, neutrophil gelatinase-associated lipocalin; BMI, body mass index; W, waist circumference; FM, fat mass; Fat%, fat percentage; SFA, subcutaneous fat area; VFA, visceral fat area

Partial correlation analysis adjusted age, gender and menopausal status (in women). * *P* < 0.05, ** *P *< 0.01

**Fig. 1:**
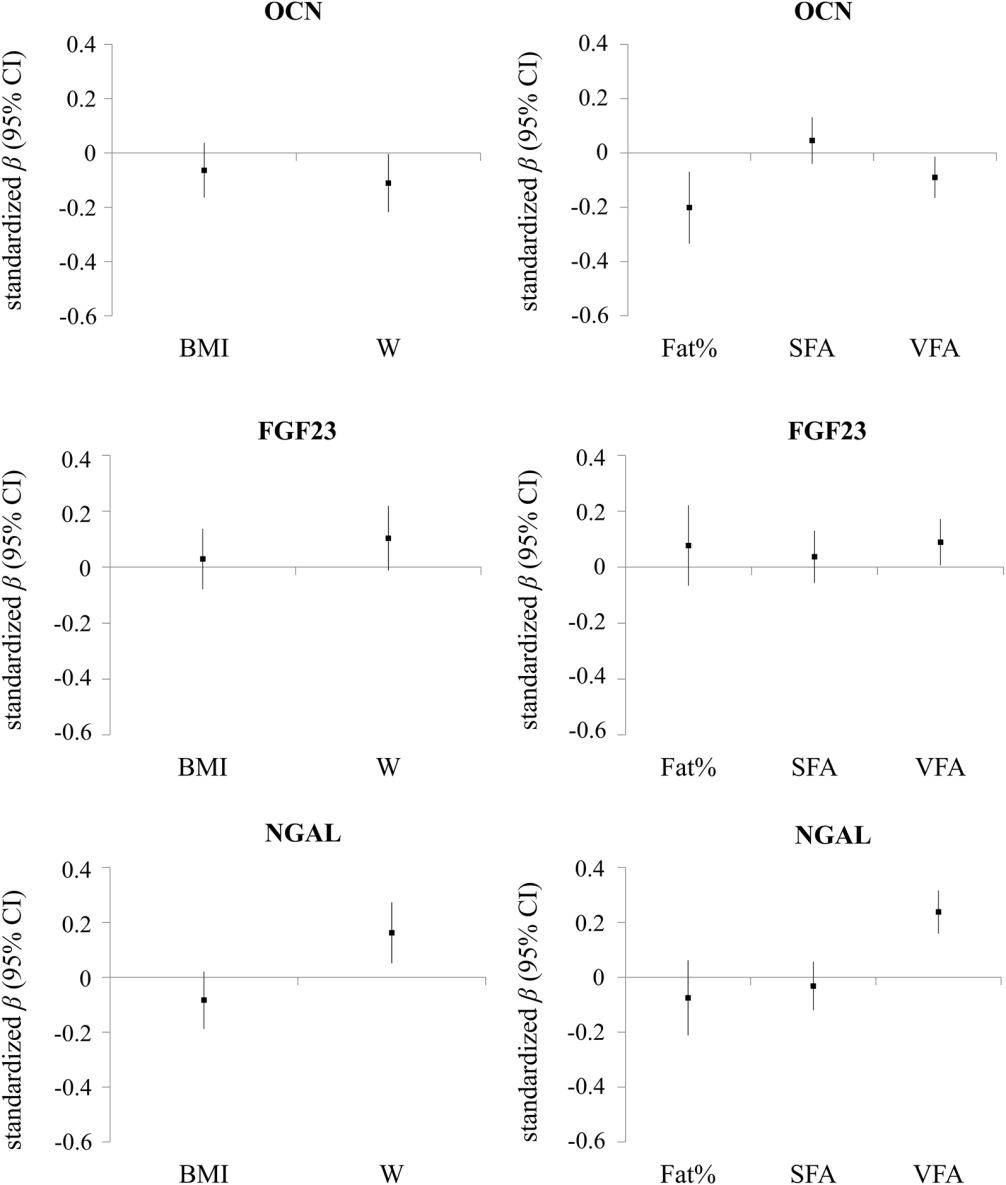
Adjusted associations of standardized adiposity measure with serum OCN, FGF23, and NGAL levels. Results were based on linear regression analyses, and expressed as standardized *β* in each bone-derived secretory factor (with 95% confidence interval) per standard deviation of adiposity measure. Adjustment was for age, gender, menopausal status (in women), smoking, SBP, DBP, HbA_1c_, HOMA-IR, TG, HDL-C, LDL-C, CRP, Ca, and eGFR

To explore the relationship between body fat parameters and serum OCN, FGF23 and NGAL levels, we performed a multivariate regression analysis in which the three bone-derived factors were used as the dependent variables and overall and abdominal adiposity indicators were used as independent variables. In model 1, W, but not BMI, was negatively correlated with serum OCN levels after adjusting for confounding variables (standardized *β* = − 0.111, *P* = 0.042). Neither BMI nor W exhibited a statistically significant correlation with serum FGF23 levels (both *P* > 0.05). Serum NGAL levels were independently and positively correlated with W, not BMI (standardized *β* = 0.162, *P* = 0.004). In model 2, fat%, SFA and VFA were the independent variables. After adjustment, serum OCN levels were negatively correlated with VFA except for fat% (standardized *β* = − 0.090, *P* = 0.021). In addition, significant positive correlations between VFA and serum FGF23 and NGAL levels were observed (standardized *β* = 0.089, *P* = 0.035; standardized *β* = 0.238, *P* < 0.001, respectively); however, the correlations with fat% and SFA did not reach statistical significance (Fig. 1).

### Association of bone-derived factors with visceral obesity

**Fig. 2:**
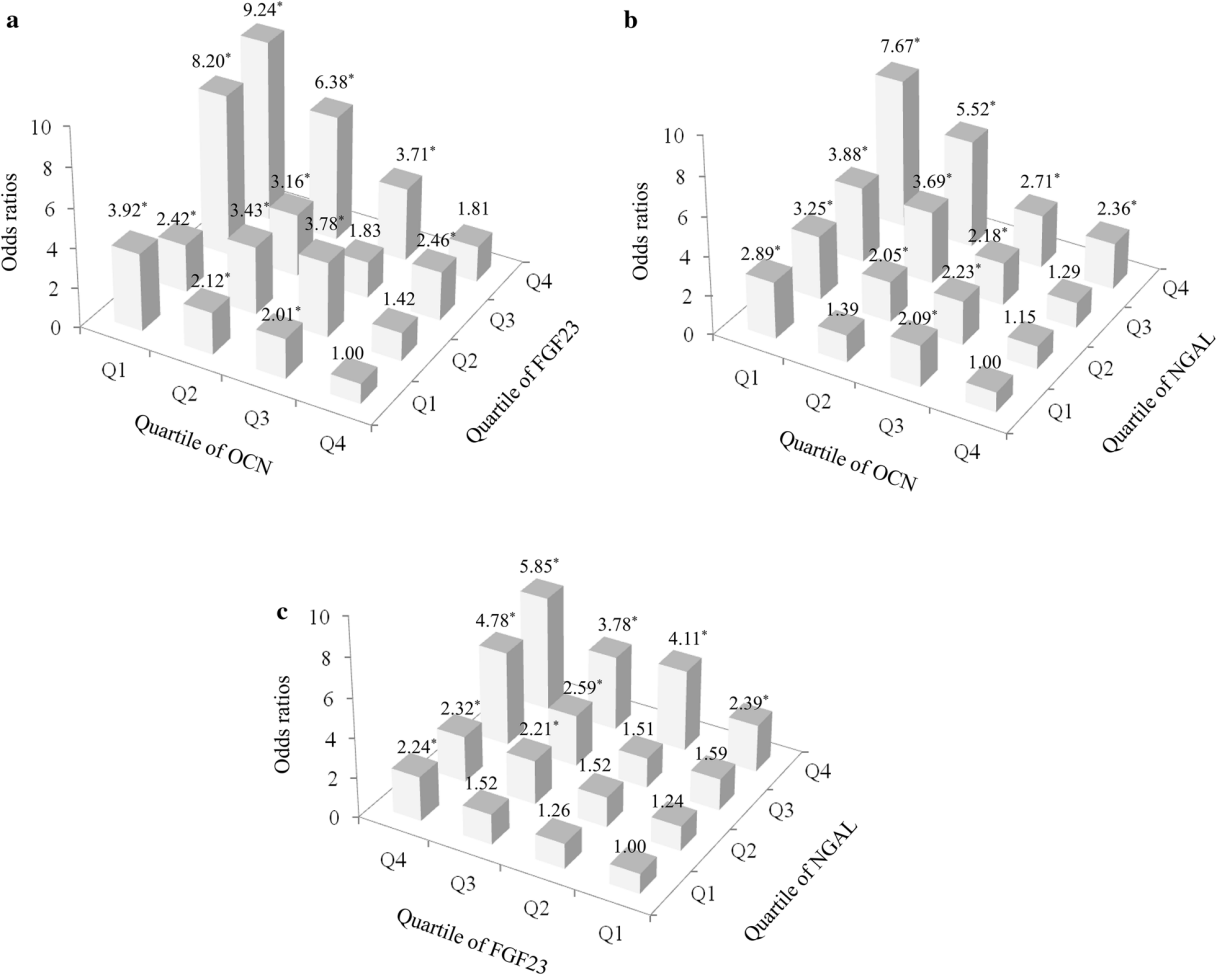
Odds ratios for visceral obesity (VFA ≥ 80 cm^2^) according to the quartiles of serum OCN and FGF23 levels (**a**), serum OCN and NGAL levels (**b**), serum FGF23 and NGAL levels (**c**). *P < 0.05

Subjects were divided into four groups according to the quartiles of each bone-derived factor to investigate the association of serum OCN, FGF23, and NGAL levels with VFA. As shown in Fig. 2, using the risk of visceral obesity in the highest quartile of OCN levels + the lowest quartile of FGF23 levels as a reference, the risk was increased 2.01- to 9.24-fold among subjects with decreased OCN levels and higher FGF23 levels (*P* < 0.05). Even in the highest quartile of OCN levels, the odds ratios (OR) for visceral obesity tended to increase as FGF23 levels increased (*P* for trend = 0.051); conversely, in the lowest quartile of FGF23 levels, the OR for visceral obesity increased as OCN levels decreased (*P* for trend < 0.001). In addition, using the risk of visceral obesity in the highest quartile of OCN levels + the lowest quartile of NGAL levels as a reference, subjects with decreased OCN levels and higher NGAL levels experienced a 2.05- to 7.67-fold risk of visceral obesity (*P* < 0.05). In the highest quartile of OCN levels, higher NGAL levels accompanied an increased OR for visceral obesity (*P* for trend = 0.015); in the lowest quartile of NGAL levels, the OR for visceral obesity also increased as the OCN levels decreased (*P* for trend = 0.007). Correspondingly, when we used the risk of visceral obesity in the lowest quartiles of both FGF23 and NGAL levels as a reference, the risk of visceral obesity was increased 2.21- to 5.85-fold with an increase in FGF23 levels and NGAL levels (*P* < 0.05). Additionally, the OR for visceral obesity increased with either higher FGF23 levels or higher NGAL levels in lowest quartile of the opposite bone-derived factor (*P* for trend = 0.010 and *P* for trend = 0.009, respectively).

Setting the presence of visceral obesity as the dependent variable, the logistic regression analysis suggested that serum OCN levels were negatively correlated with visceral obesity, whereas both serum FGF23 and NGAL levels were positively correlated (*P* < 0.001). These relationships remained significant, even in the multivariate model (*P* < 0.01). When serum OCN, FGF23 and NGAL levels were included in the multivariate model simultaneously, all of them were independently associated with visceral obesity (OCN: OR = 0.743, *P* < 0.001; FGF23: OR = 1.335, *P* < 0.001; NGAL: OR = 1.291, *P* = 0.001). Moreover, there was no interaction of serum OCN, FGF23 and NGAL levels in relation to visceral obesity (*P* > 0.05, see Table 3).

**Table 3:** Association of per 1.0 SD increase in serum OCN, FGF23 and NGAL levels with visceral obesity (VFA ≥ 80 cm^2^)

Variable	Individual models				Combined model, adjusted	
	Unadjusted		Adjusted			
	OR (95% CI)	*P* value	OR (95% CI)	*P* value	OR (95% CI)	*P* value
Total (n = 1179)						
OCN	0.647 (0.570–0.734)	< 0.001	0.727 (0.621–0.852)	< 0.001	0.743 (0.632–0.872)	< 0.001
FGF23	1.367 (1.212–1.541)	< 0.001	1.326 (1.143–1.538)	< 0.001	1.335 (1.149–1.552)	< 0.001
NGAL	1.471 (1.299–1.667)	< 0.001	1.291 (1.112–1.500)	0.001	1.291 (1.110–1.503)	0.001
Normal glucose tolerance (n = 633)						
OCN	0.659 (0.551–0.788)	< 0.001	0.745 (0.589–0.942)	0.014	0.747 (0.589–0.947)	0.016
FGF23	1.290 (1.103–1.507)	0.001	1.263 (1.033–1.545)	0.023	1.269 (1.036–1.555)	0.022
NGAL	1.487 (1.245–1.776)	< 0.001	1.253 (1.011–1.553)	0.040	1.257 (1.013–1.559)	0.037
Hyperglycaemia (n = 546)						
OCN	0.654 (0.545–0.785)	< 0.001	0.724 (0.579–0.905)	0.005	0.760 (0.605–0.955)	0.019
FGF23	1.545 (1.271–1.879)	< 0.001	1.383 (1.095–1.746)	0.006	1.383 (1.093–1.750)	0.007
NGAL	1.443 (1.205–1.728)	< 0.001	1.321 (1.065–1.639)	0.011	1.304 (1.045–1.628)	0.019

Adjustment was for age, gender, menopausal status (in women), smoking, SBP, DBP, HbA_1c_, HOMA-IR, TG, HDL-C, LDL-C, CRP, Ca, and eGFR. For the individual models, each bone-derived secretory factor was analyzed separately; for the combined model, serum OCN, FGF23, and NGAL levels were included in the model simultaneously

SD, standard deviation; OCN, osteocalcin; FGF23, fibroblast growth factor 23; NGAL, neutrophil gelatinase-associated lipocalin; VFA, visceral fat area; SBP, systolic blood pressure; DBP, diastolic blood pressure; HbA_1c_, glycated haemoglobin A_1c_; HOMA-IR, homeostasis model assessment-insulin resistance index; TG, triglyceride; HDL-C, high-density lipoprotein cholesterol; LDL-C, low-density lipoprotein cholesterol; CRP, C-reactive protein; Ca, calcium; eGFR, estimated glomerular filtration rate

### Subgroup analyses

To examine the robustness of our findings, we further conducted stratified analyses to determine the correlations between serum OCN, FGF23 and NGAL levels with the risk of visceral obesity in subgroups. Among subjects with normal glucose tolerance, the levels of the three bone-derived factors were associated with visceral obesity in the multivariate analysis after adjustment (*P* < 0.05). The independent relationships remained when the three bone-derived factors were included in the model simultaneously (OCN: OR = 0.747, *P *= 0.016; FGF23: OR = 1.269, *P* = 0.022; NGAL: OR = 1.257, *P* = 0.037). Additionally, the relationships between serum OCN, FGF23 and NGAL levels with visceral obesity still persisted among subjects with hyperglycaemia (*P* < 0.05, see Table 3).

## Discussion

As shown in the present study, the levels of the three bone-derived factors (OCN, FGF23, and NGAL) were all associated with visceral obesity; moreover, the relationships were independent of each other. The finding was further confirmed in the subgroup analyses separated by hyperglycaemia, because of the evidence that visceral fat was independently associated with hyperglycaemia regardless of obesity status [18].

### Relationship of OCN, FGF23, NGAL with visceral adiposity

Recently, concepts have arisen from the finding that bone is an important endocrine organ. The roles of serum OCN and FGF23 levels in regulating energy metabolism have led to new insights into the physiological functions of bone [8, 19, 20]. Maddaloni et al. suggested that circulating OCN + monocytes could be a marker for vascular disease in diabetic patients [21]. Our previous studies found that a decreasing trend in serum OCN levels accompanied an increase in VFA, and VFA was inversely correlated with serum OCN levels [11, 12]. Serum FGF23 levels were independently and positively correlated with the presence of lowerextremity atherosclerotic disease among Chinese patients with type 2 diabetes mellitus [22]. In addition, serum FGF23 levels were elevated in obese individuals, especially those with visceral obesity, and this relationship was not affected by hyperglycaemia [9]. We also confirmed that individuals with obesity had elevated serum NGAL levels; furthermore, serum NGAL levels were significantly positively correlated with VFA [10]. Similarly, Wang et al. observed significantly higher circulating NGAL levels in obese people than non-obese people in a Hong Kong population. Moreover, elevated NGAL levels in serum and plaques were associated with type 2 diabetes mellitus in patients with carotid artery stenosis [23]. Based on these findings, circulating NGAL levels were associated with metabolic diseases, including obesity [24–26].

Consistent with the aforementioned results, serum OCN levels were negatively correlated with adiposity indicators, while serum FGF23 and NGAL levels were both positively correlated with these indicators in the present study. Moreover, compared with overall adiposity, including BMI and fat%, the levels of the three bone-derived factors were more closely related to visceral adiposity, such as W and VFA, particularly the accurate parameter VFA.

### OCN, FGF23, NGAL in regulation of obesity

In accordance with the findings from human studies, basic research has also confirmed the protective effect of OCN on obesity. Mice lacking OCN exhibit significantly increased adiposity and tend to be obese [2]. Wild-type mice implanted with pumps that continuously deliver osteocalcin exhibit decreased body fat mass [27]. The protective role of OCN in mice is possibly mediated by an induction of adiponectin expression and improvements in insulin sensitivity, which was supported by evidence that osteocalcin improved insulin sensitivity through its division of adipocytes to increase adiponectin production, therefore, presence of osteocalcin highly favors activity of adiponectin [28]; in addition, osteocalcin improved insulin resistance by decreasing inflammation, and increasing insulin signaling and the expression of Slc2a4/GLUT4 in white adipose tissue [29]. In the present study, serum OCN levels were observed to be negatively correlated with visceral adiposity in subjects with normal glucose tolerance, suggesting that the correlations were not interfered with hyperglycaemia.

In the study by Streicher et al., mice in which FGF23 was ablated displayed reduced body weight and fat contents compared with wild type mice [8]. Adipokines directly stimulate FGF23 expression in bone, suggesting a feedback effect of adipose tissue on serum FGF23 levels [30]. However, the finding that elevated serum FGF23 levels were more closely related with visceral obesity remains to be further explored.

Recent studies have revealed that LCN-2 crosses the blood–brain barrier and suppresses appetite by binding to the melanocortin 4 receptor in the hypothalamus [4]. However, a significant increase in circulating LCN-2 levels was observed in obese mice compared to lean mice [31]. Human studies revealed that subjects with obesity had higher levels of circulating NGAL, consistent with our findings. Serum NGAL levels in high saturated fat consumers were significantly higher than those in low saturated fat consumers, even after adjusting for confounding factors [32]; in addition, an acute increase in circulating NGAL after fat overload was found in obese subjects [33]. Although the underlying mechanism for the positive correlation between NGAL and obesity remains unclear, a compensatory mechanism may be an explanation.

### Interaction between OCN, FGF23, NGAL and visceral adiposity

Although the crosstalk between bone and adipose tissue has gradually received increasing attention from researchers, no studies have been published focusing on whether the relationships of these factors with obesity are independent of each other. Strength of this study was that we identified independent relationship between serum OCN, FGF23 and NGAL levels with visceral obesity in a Chinese community-based population. The stratified analyses in OCN, FGF23 and NGAL revealed that within the same levels of one bone-derived factor, the risk of visceral obesity showed an increasing trend across the quartiles of the other two factors, respectively. Moreover, there was no interaction among the relationship between three bone-derived factors and visceral adiposity. According to these findings, we proposed that serum OCN, FGF23 and NGAL levels probably regulated energy metabolism through different pathways. In obese patients, more attention should be paid to their serum concentrations of bone-derived factors, which are expected to provide new insight into targets for therapeutic intervention of obesity.

We acknowledge that our study has several limitations. This study was unable to clarify the causal relationships between serum OCN, FGF23, and NGAL levels with body fat parameters, particularly with VFA. Therefore, the relevant effects of serum OCN, FGF23 and NGAL levels on obesity must be further confirmed in large-scale prospective studies and mechanistic studies. We measured only serum total OCN due to the lack of an automated assay to determine its under-carboxylated form, though they were closely related. In addition, bone mineral density was not assessed in this study, which could be included in the adjustment in future studies.

## Conclusions

In conclusion, serum OCN levels were inversely correlated with body fat parameters, while serum FGF23 and NGAL levels were positively correlated. Compared with overall adiposity, visceral adiposity had closer relationships with the levels of the three bone-derived factors. The relationships between the levels of the three factors and visceral obesity were independent of each other, suggesting that hormones secreted by bone probably participate in the regulation of energy metabolism through different pathways.

## Additional files

**Additional file 1: Table S1.** Clinical characteristics of the study subjects (with and without overweight/obesity).

**Additional file 2: Table S2.** Clinical characteristics of the study subjects (with and without hyperglycaemia).

## Abbreviations

BMI: body mass index
CRP: C-reactive protein
eGFR: estimated glomerular filtration rate
Fat%: fat percentage
FGF: fibroblast growth factor
FM: fat mass
FPG: fasting plasma glucose
HbA_1c_: glycated haemoglobin A_1c_
HDL-C: high-density lipoprotein cholesterol
HOMA**-**IR: homeostasis model assessment-insulin resistance index
LCN-2: lipocalin-2
LDL-C: low-density lipoprotein cholesterol
NGAL: neutrophil gelatinase-associated lipocalin
OCN: osteocalcin
OR: odds ratios
SFA: subcutaneous fat area
TG: triglyceride
VFA: visceral fat area
W: waist circumference
2hPG: 2-h plasma glucose

## References

[1] Han Y, You X, Xing W, Zhang Z, Zou W (2018). Paracrine and endocrine actions of bone-the functions of secretory proteins from osteoblasts, osteocytes, and osteoclasts. Bone Res..

[2] Lee NK, Sowa H, Hinoi E, Ferron M, Ahn JD, Confavreux C (2007). Endocrine regulation of energy metabolism by the skeleton. Cell.

[3] David V, Dai B, Martin A, Huang J, Han X, Quarles LD (2013). Calcium regulates FGF-23 expression in bone. Endocrinology.

[4] Mosialou I, Shikhel S, Liu JM, Maurizi A, Luo N, He Z (2017). MC4R-dependent suppression of appetite by bone-derived lipocalin 2. Nature.

[5] Zanatta LC, Boguszewski CL, Borba VZ, Kulak CA (2014). Osteocalcin, energy and glucose metabolism. Arq Bras Endocrinol Metabol..

[6] Martin A, David V, Quarles LD (2012). Regulation and function of the FGF23/klotho endocrine pathways. Physiol Rev.

[7] Reid IR (2010). Fat and bone. Arch Biochem Biophys.

[8] Streicher C, Zeitz U, Andrukhova O, Rupprecht A, Pohl E, Larsson TE (2012). Long-term Fgf23 deficiency does not influence aging, glucose homeostasis, or fat metabolism in mice with a nonfunctioning vitamin D receptor. Endocrinology.

[9] Hu X, Ma X, Luo Y, Xu Y, Xiong Q, Pan X (2016). Associations of serum fibroblast growth factor 23 levels with obesity and visceral fat accumulation. Clin Nutr.

[10] Luo Y, Ma X, Pan X, Xu Y, Xiong Q, Xiao Y (2016). Serum lipocalin-2 levels are positively associated with not only total body fat but also visceral fat area in Chinese men. Medicine (Baltimore).

[11] Luo Y, Ma X, Hao Y, Xu Y, Xiong Q, Tang J (2015). Association between serum osteocalcin level and visceral obesity in Chinese postmenopausal women. Clin Endocrinol (Oxf).

[12] Bao Y, Ma X, Yang R, Wang F, Hao Y, Dou J (2013). Inverse relationship between serum osteocalcin levels and visceral fat area in Chinese men. J Clin Endocrinol Metab.

[13] Levey AS, Stevens LA, Schmid CH, Zhang YL, Castro AF, Feldman HI (2009). CKD-EPI (Chronic Kidney Disease Epidemiology Collaboration). A new equation to estimate glomerular filtration rate. Ann Intern Med.

[14] Zhou M, Ma X, Li H, Pan X, Tang J, Gao Y (2009). Serum osteocalcin concentrations in relation to glucose and lipid metabolism in Chinese individuals. Eur J Endocrinol.

[15] World Health Organization (2000). Obesity: preventing and managing the global epidemic. Report of a WHO consultation. World Health Organ Tech Rep Serv.

[16] Bao Y, Lu J, Wang C, Yang M, Li H, Zhang X (2008). Optimal waist circumference cutoffs for abdominal obesity in Chinese. Atherosclerosis..

[17] Department of Noncommunicable Disease Surveillance (1999). Report of a WHO consultation: definition, diagnosis and classification of diabetes mellitus and its complication. Part 1: Diagnosis and classification of diabetes mellitus.

[18] Lv X, Zhou W, Sun J, Lin R, Ding L, Xu M (2017). Visceral adiposity is significantly associated with type 2 diabetes in middle-aged and elderly Chinese women: a cross-sectional study. J Diabetes..

[19] Ferron M, Lacombe J (2014). Regulation of energy metabolism by the skeleton: osteocalcin and beyond. Arch Biochem Biophys.

[20] Ferron M, McKee MD, Levine RL, Ducy P, Karsenty G (2012). Intermittent injections of osteocalcin improve glucose metabolism and prevent type 2 diabetes in mice. Bone.

[21] Maddaloni E, Xia Y, Park K, D’Eon S, Tinsley LJ, St-Louis R (2017). High density lipoprotein modulates osteocalcin expression in circulating monocytes: a potential protective mechanism for cardiovascular disease in type 1 diabetes. Cardiovasc Diabetol..

[22] He X, Hu X, Ma X, Su H, Ying L, Peng J (2017). Elevated serum fibroblast growth factor 23 levels as an indicator of lower extremity atherosclerotic disease in Chinese patients with type 2 diabetes mellitus. Cardiovasc Diabetol..

[23] Eilenberg W, Stojkovic S, Piechota-Polanczyk A, Kaider A, Kozakowski N, Weninger WJ (2017). Neutrophil gelatinase associated lipocalin (NGAL) is elevated in type 2 diabetics with carotid artery stenosis and reduced under metformin treatment. Cardiovasc Diabetol..

[24] Wang Y, Lam KS, Kraegen EW, Sweeney G, Zhang J, Tso AW (2007). Lipocalin-2 is an inflammatory marker closely associated with obesity, insulin resistance, and hyperglycemia in humans. Clin Chem.

[25] Wu G, Li H, Fang Q, Jiang S, Zhang L, Zhang J (2014). Elevated circulating lipocalin-2 levels independently predict incident cardiovascular events in men in a population-based cohort. Arterioscler Thromb Vasc Biol.

[26] Xiang Y, Zhou P, Li X, Huang G, Liu Z, Xu A (2011). Heterogeneity of altered cytokine levels across the clinical spectrum of diabetes in China. Diabetes Care.

[27] Ferron M, Hinoi E, Karsenty G, Ducy P (2008). Osteocalcin differentially regulates beta cell and adipocyte gene expression and affects the development of metabolic diseases in wild-type mice. Proc Natl Acad Sci USA.

[28] Zhang Y, Zhou P, Kimondo JW (2012). Adiponectin and osteocalcin: relation to insulin sensitivity. Biochem Cell Biol.

[29] Guedes JAC, Esteves JV, Morais MR, Zorn TM, Furuya DT (2018). Osteocalcin improves insulin resistance and inflammation in obese mice: participation of white adipose tissue and bone. Bone.

[30] Tsuji K, Maeda T, Kawane T, Matsunuma A, Horiuchi N (2010). Leptin stimulates fibroblast growth factor 23 expression in bone and suppresses renal 1alpha,25-dihydroxyvitamin D3 synthesis in leptin-deficient mice. J Bone Miner Res.

[31] Yan QW, Yang Q, Mody N, Graham TE, Hsu CH, Xu Z (2007). The adipokine lipocalin 2 is regulated by obesity and promotes insulin resistance. Diabetes.

[32] Na GY, Yoon SR, An J, Yeo R, Song J, Jo MN (2017). The relationship between circulating neutrophil gelatinase-associated lipocalin and early alteration of metabolic parameters is associated with dietary saturated fat intake in non-diabetic Korean women. Endocr J.

[33] Moreno-Navarrete JM, Manco M, Ibáñez J, García-Fuentes E, Ortega F, Gorostiaga E (2010). Metabolic endotoxemia and saturated fat contribute to circulating NGAL concentrations in subjects with insulin resistance. Int J Obes(Lond).

