# Supplementary material for: Correlations between serum concentration of three bone-derived factors and obesity and visceral fat accumulation in a cohort of middle aged men and women
Source: Cardiovasc Diabetol. 2018 Nov 13;17:143. doi: 10.1186/s12933-018-0786-9 (PMC6233377; doi:10.1186/s12933-018-0786-9)
Supplement: Supplementary file 1 — Additional file 1: Table S1. Clinical characteristics of the study subjects (with and without overweight/obesity). [file 12933_2018_786_MOESM1_ESM.doc]

**Additional file 1: Table S1** Clinical Characteristics of the study subjects

| Variables | Non-overweight/obesity (n = 852) |  | Overweight/obesity (n = 327) |  | *P* Value |
| --- | --- | --- | --- | --- | --- |
| Age (years) | 59.8 ± 6.2 |  | 58.9 ± 6.3 |  | 0.037 |
| Men, n (%) | 333 (39.1) |  | 132 (40.4) |  | 0.687 |
| BMI (kg/m^2^) | 22.4 ± 2.1 |  | 27.4 ± 2.0 |  | < 0.001 |
| W (cm) | 80.0 (74.5–84.0) |  | 92.0 (86.5–96.0) |  | < 0.001 |
| FM (kg) | 15.5 (12.6–18.3) |  | 23.9 (20.9–27.2) |  | < 0.001 |
| Fat% | 26.6 (21.0–31.9) |  | 36.1 (26.5–39.0) |  | < 0.001 |
| SFA (cm^2^) | 152.0 (119.5–189.5) |  | 222.4 (179.6–275.9) |  | < 0.001 |
| **VFA** (cm^2^) | 68.1 (46.7–88.9) |  | 108.3 (84.6–132.7) |  | < 0.001 |
| SBP (mmHg) | 126.0 (115.0–138.0) |  | 133.0 (122.0–146.0) |  | < 0.001 |
| DBP (mmHg) | 76.0 (69.0–82.0) |  | 80.0 (72.0–87.0) |  | < 0.001 |
| FPG (mmol/L) | 5.7 (5.3–6.0) |  | 5.8 (5.5–6.3) |  | < 0.001 |
| 2hPG (mmol/L) | 7.0 (5.7–8.2) |  | 7.6 (6.0–9.4) |  | < 0.001 |
| HbA_1c_ (%) | 5.6 (5.4–5.9) |  | 5.7 (5.5–6.0) |  | 0.044 |
| **Fasting insulin** (mu/L) | 7.6 (5.5–10.3) |  | 11.6 (8.5–16.0) |  | < 0.001 |
| HOMA-IR | 1.9 (1.4–2.7) |  | 3.1 (2.2–4.3) |  | < 0.001 |
| TC (mmol/L) | 5.5 ± 1.0 |  | 5.5 ± 1.0 |  | 0.829 |
| TG (mmol/L) | 1.2 (0.9–1.8) |  | 1.6 (1.2–2.2) |  | < 0.001 |
| HDL-C (mmol/L) | 1.5 (1.2–1.7) |  | 1.3 (1.1–1.6) |  | < 0.001 |
| LDL-C (mmol/L) | 3.3 ± 0.8 |  | 3.4 ± 0.8 |  | 0.009 |
| CRP (mg/L) | 0.7 (0.4–1.4) |  | 1.3 (0.7–2.5) |  | < 0.001 |
| Ca (mmol/L) | 2.4 (2.4–2.5) |  | 2.4 (2.3–2.5) |  | 0.021 |
| eGFR (mL/min/1.73 m^2^) | 97.1 (90.7–103.0) |  | 96.6 (89.8–102.1) |  | 0.641 |
| OCN (ng/mL) | 20.4 (16.1–25.3) |  | 18.7 (15.2–22.5) |  | < 0.001 |
| FGF23 (pg/mL) | 34.2 (27.5–40.8) |  | 36.3 (28.3–44.2) |  | < 0.001 |
| NGAL (ng/mL) | 44.3 (31.0–60.7) |  | 47.0 (32.4–65.0) |  | 0.085 |
| Smoking, n (%) | 173 (20.3) |  | 68 (20.8) |  | 0.852 |
| Visceral obesity**, n (%)** | 303 (35.6) |  | 261 (79.8) |  | < 0.001 |
| Hyperglycaemia**, n (%)** | 364 (42.7) |  | 182 (55.7) |  | < 0.001 |

**Abbreviation: BMI, body mass index; W, waist circumference; FM, fat mass; Fat %, fat percentage; SFA, subcutaneous fat area; VFA, visceral fat area; SBP, systolic blood pressure; DBP, diastolic blood pressure; FPG, fasting plasma glucose; 2hPG, 2-h plasma glucose; HbA_1c_, glycated haemoglobin; HOMA-IR, homeostasis model assessment-insulin resistance index; TC, total cholesterol; TG, triglyceride; HDL-C, high-density lipoprotein cholesterol; LDL-C, low-density lipoprotein cholesterol; CRP, C-reactive protein; Ca, calcium; eGFR, estimated glomerular filtration rate; OCN, osteocalcin; FGF23, fibroblast growth factor 23;** NGAL**, neutrophil gelatinase-associated lipocalin.**
