# Supplementary material for: Correlations between serum concentration of three bone-derived factors and obesity and visceral fat accumulation in a cohort of middle aged men and women
Source: Cardiovasc Diabetol. 2018 Nov 13;17:143. doi: 10.1186/s12933-018-0786-9 (PMC6233377; doi:10.1186/s12933-018-0786-9)
Supplement: Supplementary file 2 — Additional file 2: Table S2. Clinical characteristics of the study subjects (with and without hyperglycaemia). [file 12933_2018_786_MOESM2_ESM.doc]

**Additional file 2: Table S2** Clinical Characteristics of the study subjects

| Variables | Non-hyperglycaemia (n = 633) |  | Hyperglycaemia (n = 546) |  | *P* Value |
| --- | --- | --- | --- | --- | --- |
| Age (years) | 58.7 ± 6.2 |  | 60.5 ± 6.1 |  | < 0.001 |
| Men, n (%) | 228 (36.0) |  | 237 (43.4) |  | 0.010 |
| BMI (kg/m^2^) | 23.4 ± 3.0 |  | 24.3 ± 3.1 |  | < 0.001 |
| W (cm) | 81.0 (75.3–87.0) |  | 84.5 (79.0–91.0) |  | < 0.001 |
| FM (kg) | 17.1 (13.6–20.9) |  | 17.9 (14.3–22.1) |  | 0.002 |
| Fat% | 28.0 (22.5–33.3) |  | 28.3 (22.8–34.8) |  | 0.126 |
| SFA (cm^2^) | 165.8 (127.4–212.0) |  | 171.0 (134.5–223.0) |  | 0.047 |
| **VFA** (cm^2^) | 71.3 (47.5–94.9) |  | 85.6 (63.8–113.7) |  | < 0.001 |
| SBP (mmHg) | 125.0 (114.0–137.0) |  | 131.0 (120.8–144.0) |  | < 0.001 |
| DBP (mmHg) | 75.0 (68.0–82.5) |  | 78.0 (72.0–85.0) |  | < 0.001 |
| FPG (mmol/L) | 5.5 (5.2–5.8) |  | 6.1 (5.7–6.6) |  | < 0.001 |
| 2hPG (mmol/L) | 6.1 (5.1–6.9) |  | 8.8 (7.9–10.2) |  | < 0.001 |
| HbA_1c_ (%) | 5.6 (5.4–5.9) |  | 5.8 (5.5–6.1) |  | < 0.001 |
| **Fasting insulin** (mu/L) | 7.6 (5.5–10.3) |  | 9.9 (7.0–14.0) |  | < 0.001 |
| HOMA-IR | 1.8 (1.3–2.6) |  | 2.7 (1.9–4.0) |  | < 0.001 |
| TC (mmol/L) | 5.4 ± 1.0 |  | 5.6 ± 1.0 |  | 0.001 |
| TG (mmol/L) | 1.2 (1.0–1.8) |  | 1.5 (1.1–2.2) |  | < 0.001 |
| HDL-C (mmol/L) | 1.5 (1.2–1.7) |  | 1.4 (1.2–1.6) |  | < 0.001 |
| LDL-C (mmol/L) | 3.2 ± 0.8 |  | 3.4 ± 0.8 |  | < 0.001 |
| CRP (mg/L) | 0.7 (0.4–1.4) |  | 1.1 (0.5–2.1) |  | < 0.001 |
| Ca (mmol/L) | 2.4 (2.3–2.5) |  | 2.4 (2.3–2.5) |  | 0.923 |
| eGFR (mL/min/1.73 m^2^) | 97.5 (90.7–103.1) |  | 96.2 (90.3–102.3) |  | 0.130 |
| OCN (ng/mL) | 20.5 (16.5–25.2) |  | 18.9 (15.4–24.1) |  | 0.003 |
| FGF23 (pg/mL) | 35.0 (28.3–41.7) |  | 34.2 (28.3–41.8) |  | 0.981 |
| NGAL (ng/mL) | 45.1 (31.6–59.8) |  | 45.0 (30.9–64.9) |  | 0.340 |
| Smoking, n (%) | 131 (20.7) |  | 110 (20.1) |  | 0.816 |
| Overweight/Obesity**, n (%)** | 145 (22.9) |  | 182 (33.3) |  | < 0.001 |
| Visceral obesity**, n (%)** | 251 (39.7) |  | 313 (57.3) |  | < 0.001 |

**Abbreviation: BMI, body mass index; W, waist circumference; FM, fat mass; Fat %, fat percentage; SFA, subcutaneous fat area; VFA, visceral fat area; SBP, systolic blood pressure; DBP, diastolic blood pressure; FPG, fasting plasma glucose; 2hPG, 2-h plasma glucose; HbA_1c_, glycated haemoglobin; HOMA-IR, homeostasis model assessment-insulin resistance index; TC, total cholesterol; TG, triglyceride; HDL-C, high-density lipoprotein cholesterol; LDL-C, low-density lipoprotein cholesterol; CRP, C-reactive protein; Ca, calcium; eGFR, estimated glomerular filtration rate; OCN, osteocalcin; FGF23, fibroblast growth factor 23;** NGAL**, neutrophil gelatinase-associated lipocalin.**
